# Analysis of risk factors for depression in peritoneal dialysis patients and establishment of a risk nomogram model

**DOI:** 10.1016/j.clinsp.2025.100600

**Published:** 2025-02-13

**Authors:** Ming Yang, Xinhai Tang, Yehua Fang

**Affiliations:** a Department of Nephrology, Zhuzhou Central Hospital, Zhuzhou City, Hunan Province, PR China; b Department of Clinical Psychology, Zhuzhou Central Hospital, Zhuzhou City, Hunan Province, PR China

**Keywords:** Peritoneal dialysis, Depression, Risk factors, Nomogram model

## Abstract

**Objective:**

This study aims to analyze the risk factors for depression in peritoneal dialysis patients and to develop a predictive nomogram model for assessing these risks.

**Methods:**

A total of 326 peritoneal dialysis patients treated between August 2021 and December 2023 were selected as the training set. These patients were categorized into a non-depression group (229 cases) and a depression group (97 cases) based on the presence of depression. Additionally, 104 peritoneal dialysis patients from the same period were selected as the validation set. Clinical data were collected from all patients for analysis.

**Results:**

The depression group had higher proportions of female patients, non-employed individuals, those with a per capita monthly income of <2000-yuan, cardiovascular complications, cerebrovascular complications, and sleep disorders compared to the non-depression group. Additionally, the levels of hemoglobin and blood uric acid were lower in the depression group than in the non-depression group (*p* < 0.05). Gender, work status, per capita monthly income, cardiovascular complications, cerebrovascular complications, and sleep disorders are risk factors for depression in peritoneal dialysis patients (*p* < 0.05), while hemoglobin and blood uric acid are protective factors for depression in peritoneal dialysis patients (*p* < 0.05). Calibration curve analysis results showed that the predicted probability was basically consistent with the actual incidence rate. The results of the Decision Curve Analysis (DCA) demonstrated that the nomogram model developed in this study has strong clinical applicability.

**Conclusion:**

The nomogram model for predicting depression in peritoneal dialysis patients, which incorporates factors such as gender, work status, per capita monthly income, cardiovascular complications, cerebrovascular complications, sleep disorders, hemoglobin levels, and blood uric acid levels, demonstrates excellent calibration and discrimination. Additionally, it has high clinical applicability.

## Highlights

• Risk factors for depression in peritoneal dialysis patients identified.
• Nomogram predicts depression risk in peritoneal dialysis patients.
• Gender, income, and comorbidities influence depression risk in dialysis.
• Low hemoglobin and uric acid levels are linked to higher depression risk.
• Developed nomogram shows strong clinical applicability and accuracy.

## Introduction

Peritoneal dialysis is an important method of replacement therapy for end-stage renal disease, with minimal impact on hemodynamics, preservation of residual kidney function, and reduced cardiovascular risk.^1^^,^^2^ However, patients undergoing long-term dialysis treatment often face challenges such as limited mobility, high treatment costs, and significant physical and mental distress. These factors can lead to negative emotions, including anxiety and depression, which not only diminish the effectiveness of dialysis treatment but also severely impact the quality of life for patients and their families.^3^^,^^4^ Tian et al.^5^ found that the prevalence of depression among dialysis patients ranges from 13.1 % to 76.3 %. Currently, there is a lack of effective tools for predicting the risk of depression in peritoneal dialysis patients. Previous studies have identified employment status, sleep disorders, comorbidity scores, and chronic nephritis as independent risk factors for depression in patients undergoing maintenance hemodialysis.^6^, ^7^, ^8^ However, the above studies are not suitable for predicting the occurrence of depression in peritoneal dialysis patients and target different study populations. In this study, the goal is to develop an accurate and simple predictive tool to estimate the risk of depression in patients undergoing peritoneal dialysis.

## Subjects and methods

### *Study subjects*

A total of 326 peritoneal dialysis patients, treated between August 2021 and December 2023, were selected for the training set. These patients were divided into a non-depression group (229 cases, with a depression self-rating scale score of < 53) and a depression group (97 cases, with a depression self-rating scale score of ≥ 53). Additionally, 104 peritoneal dialysis patients from the same period were selected as the validation set. This study adheres to ethical standards, involves informed consent from all participants, and does not infringe on patient privacy.

**Fig. 1:**
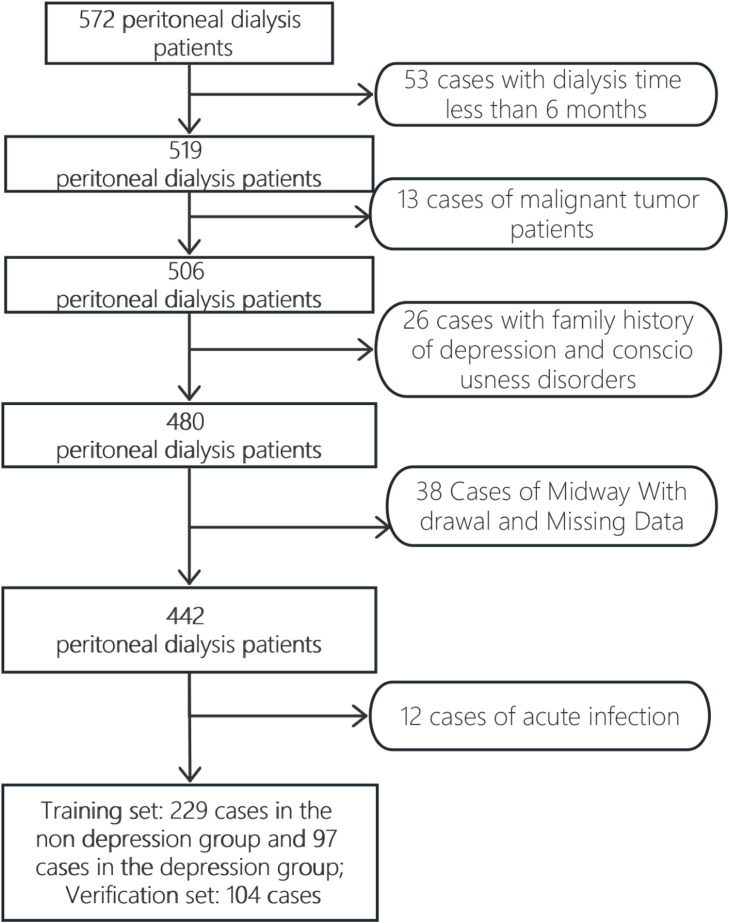
Case collection process diagram.

Inclusion criteria: 1) Patients undergoing peritoneal dialysis for >6-months, with a stable condition and an expected survival time of over 1-year. According to the 11th edition of the International Classification of Diseases,^9^ a score of ≥ 53 on the self-rating depression scale is defined as indicative of depression. 2) Patients are able to communicate normally, with no cognitive impairments. 3) Patients aged 18-years or older. 4) Patients with relatively high compliance and ability to cooperate with treatment. Exclusion criteria: 1) Patients with acute infections or malignant tumors. 2) Patients who drop out midway or have missing key information. 3) Patients with a history of organic mental disorders, schizophrenia, schizotypal disorder, neurotic disorders, stress-related disorders, and somatoform disorders. 4) Patients with a family history of depression or previous depressive symptoms. See Fig. 1 for the case collection flowchart.

### *Clinical data collection*

According to the National Bureau of Statistics' report “2018 National Time Use Survey Bulletin”, collects data on patients' age (some patients over 60-years old are engaged in jobs such as landscaping maintenance, community security, warehouse management, and community cleaning), gender, education level, employment status, marital status, and average monthly income (with below 2000 yuan categorized as low income, and 2000 to 5000 yuan categorized as middle income). Most participants in this study fall into the 2000 to 5000 yuan range, with fewer individuals earning over 5000 yuan. Therefore, the study combined the groups earning between 2000 and 5000 yuan and used “above 2000 yuan” to represent the income categories. Collect data on payment method for expenses, alcohol intake, smoking, hypertension, diabetes, cardiovascular complications, cerebrovascular complications, daily dialysis frequency, dialysis duration, primary disease, and sleep disorders (defined as changes in sleep duration and/or quality caused by various factors, impacting daytime social functioning; a sleep disorder can be diagnosed if the patient reports a decrease or increase in sleep quantity, exhibits abnormal behaviors during sleep, or experiences changes in the sleep-wake rhythm), hemoglobin, blood uric acid, albumin, protein catabolic rate (calculated as [pre-dialysis blood urea nitrogen-post-dialysis blood urea nitrogen] × [0.045 / number of days between two blood samples]), urea clearance rate (KT/V) of peritoneal dialysis patients.

### *Statistical analysis*

Data processing was performed using SPSS 25.0 software. The χ^2^ test was used for comparing categorical data [n (%)], and the *t*-test was used for comparing continuous data (mean ± SD). Logistic regression analysis was used to screen risk factors for depression in peritoneal dialysis patients, and R 4.0.2 software and the rms package were used to draw the risk nomogram for the occurrence of depression in peritoneal dialysis patients. The Hosmer-Lemeshoe (H-L) goodness-of-fit test evaluated the fitting degree of the nomogram. The ROC curve and calibration curve were used to assess the discrimination and calibration of the nomogram, respectively, while the DCA curve was used to evaluate the clinical utility of the nomogram; *p* < 0.05 was considered statistically significant.

## Results

### *Comparison of clinical data between the validation and training sets*

There were no statistically significant differences in age, gender, education, employment status, marital status, average monthly income, payment method for expenses, alcohol intake, smoking, hypertension, diabetes, cardiovascular complications, cerebrovascular complications, daily dialysis frequency, dialysis duration, primary disease, sleep disorders, hemoglobin, blood uric acid, albumin, protein catabolic rate, KT/V between the validation set and the training set (*p* > 0.05). See Table 1.

**Table 1:** Comparison of clinical data between validation set and training set [n (%)/(±)].

Items	Validation set (*n* = 104)	Training set (*n* = 326)	*t*/χ^2^	p
Age (years)			0.087	0.957
< 45	24 (23.08)	73 (22.39)		
45∼60	38 (36.54)	116 (35.58)		
> 60	42 (40.38)	137 (42.03)		
Gender, n (%)			1.274	0.259
Male	76 (73.08)	219 (67.18)		
Female	28 (26.92)	107 (32.82)		
Education, n (%)			0.332	0.954
Primary school and below	12 (11.54)	36 (11.04)		
Middle school	22 (21.15)	62 (19.02)		
Vocational and high schools	41 (39.42)	130 (39.88)		
College degree or above	29 (27.89)	98 (30.06)		
Working condition, n (%)			0.513	0.474
Workforce	79 (75.96)	236 (72.39)		
Non workforce	25 (24.04)	90 (27.64)		
Marital status, n (%)			0.163	0.687
Married	85 (81.73)	272(83.44)		
Unmarried	19 (18.27)	54(16.56)		
Monthly income (yuan)			2.429	0.119
≥ 2000	78 (75.00)	218 (66.87)		
< 2000	26 (25.00)	108 (33.13)		
Fee payment method, n (%)			0.806	0.668
Provincial and municipal medical insurance	45 (43.27)	131 (40.19)		
New rural cooperative medical insurance	48 (46.15)	165 (50.61)		
No medical insurance	11 (10.58)	30 (9.20)		
Alcohol intake, n (%)			0.163	0.687
No	85 (81.73)	272 (83.44)		
Yes	19 (18.27)	54 (16.56)		
Smoke, n (%)			1.113	0.291
No	38 (36.54)	101 (30.98)		
Yes	66 (63.46)	225 (69.02)		
Hypertension, n (%)			0.177	0.674
No	77 (74.04)	248 (76.07)		
Yes	27 (25.96)	78 (23.93)		
Diabetes, n (%)			1.383	0.240
No	66 (63.46)	227 (69.63)		
Yes	38 (36.54)	99 (30.37)		
Angiocardiopathy, n (%)			1.398	0.237
No	79 (75.96)	265 (81.29)		
Yes	25 (24.04)	61 (18.71)		
Cerebrovascular, n (%)			0.812	0.368
No	96 (92.31)	291 (89.26)		
Yes	8 (7.69)	35 (10.74)		
Daily dialysis frequency (times)			0.127	0.721
< 3	48 (46.15)	157 (48.16)		
≥ 3	56 (53.85)	169 (51.84)		
dialysis time (year)			1.222	0.269
< 2	86 (82.69)	253 (77.61)		
≥ 2	18 (17.31)	73 (22.39)		
Primary disease, n (%)			0.980	0.806
Glomerulonephritis	49 (47.12)	166 (50.92)		
Hypertensive nephropathy	18 (17.31)	55 (16.87)		
Diabetic nephropathy	25 (24.04)	77 (23.62)		
Anaphylatic purpura nephritis	12 (11.53)	28 (8.59)		
Sleep disorders, n (%)			0.772	0.380
No	75 (72.12)	249 (76.38)		
Yes	29 (27.88)	77 (23.62)		
Hemoglobin (g/L)	106.71±17.32	104.30±16.35	1.290	0.198
Blood uric acid (μmoL/L)	412.05±80.40	409.92±83.55	0.228	0.819
Albumin (g/L)	33.92±4.96	33.77±4.87	0.272	0.786
Protein breakdown metabolic rate (%)	0.71±0.25	0.70±0.24	0.366	0.714
KT/V	1.07±0.22	1.09±0.24	0.755	0.451

### *Univariate analysis of depression in the training set of peritoneal dialysis patients*

There were no significant differences in age, education, marital status, payment method for expenses, alcohol intake, smoking, hypertension, diabetes, daily dialysis frequency, dialysis duration, primary disease, albumin, protein catabolic rate, KT/V between the two groups (*p* > 0.05). The depression group had a higher proportion of females, unemployed individuals, those with an average monthly income below 2000 Yuan, and those with cardiovascular complications, cerebrovascular complications, and sleep disorders. Additionally, levels of hemoglobin and blood uric acid were lower in the depression group compared to the non-depression group (*p* < 0.05). See Table 2.

**Table 2:** Univariate analysis of depression in peritoneal dialysis patients in the training set [n (%)/ ($\bar{x}$±s)].

**Items**	**No depression group (*n*=229)**	**Depression group (*n*=97)**	***t*/χ^2^**	**p**
Age (years)			0.422	0.810
< 45	50 (21.83)	23 (23.71)		
45∼60	84 (36.68)	32 (32.99)		
> 60	95 (41.49)	42 (43.30)		
Gender, n (%)			38.860	0.000
Male	178 (77.73)	41 (42.27)		
Female	51 (22.27)	56 (57.73)		
Education, n (%)			0.911	0.823
Primary school and below	27 (11.79)	9 (9.28)		
Middle school	43 (18.78)	19 (19.59)		
Vocational and high schools	93 (40.61)	37 (38.14)		
College degree or above	66 (28.82)	32 (32.99)		
Working condition, n (%)			33.069	0.000
Workforce	187 (81.66)	49 (50.52)		
Non workforce	42 (18.34)	48 (49.48)		
Marital status, n (%)			0.397	0.529
Married	193 (84.28)	79 (81.44)		
Unmarried	36 (15.72)	18 (18.56)		
Monthly income (yuan)			26.142	0.000
≥ 2000	173 (75.55)	45 (46.39)		
< 2000	56 (24.45)	52 (53.61)		
Fee payment method, n (%)			1.002	0.606
Provincial and municipal medical insurance	91 (39.74)	40 (41.24)		
New rural cooperative medical insurance	119 (51.97)	46 (47.42)		
No medical insurance	19 (8.29)	11 (11.34)		
Alcohol intake, n (%)			1.642	0.200
No	195 (85.15)	77 (79.38)		
Yes	34 (14.85)	20 (20.62)		
Smoke, n (%)			0.289	0.591
No	73 (31.88)	28 (28.87)		
Yes	156 (68.12)	69 (71.13)		
Hypertension, n (%)			0.628	0.428
No	177 (77.29)	71 (73.20)		
Yes	52 (22.71)	26 (26.80)		
Diabetes, n (%)			0.147	0.701
No	158 (69.90)	69 (71.13)		
Yes	71 (31.00)	28 (28.87)		
Angiocardiopathy, n (%)			34.283	0.000
No	205 (89.52)	60 (61.86)		
Yes	24 (10.48)	37 (38.14)		
Cerebrovascular, n (%)			20.556	0.000
No	216 (94.32)	75 (77.32)		
Yes	13 (5.68)	22 (22.68)		
Daily dialysis frequency (times)			0.030	0.862
< 3	111 (48.47)	46 (47.42)		
≥ 3	118 (51.53)	51 (52.58)		
Dialysis time (year)			1.546	0.214
< 2	182 (79.48)	71 (73.20)		
≥ 2	47 (20.52)	26 (26.80)		
Primary disease, n (%)			0.349	0.951
Glomerulonephritis	116 (50.66)	50 (51.55)		
Hypertensive nephropathy	38 (16.59)	17 (17.53)		
Diabetic nephropathy	56 (24.45)	21 (21.65)		
Anaphylatic purpura nephritis	19 (8.30)	9 (9.27)		
Sleep disorders, n (%)			39.692	0.000
No	197 (86.03)	52 (53.61)		
Yes	32 (13.97)	45 (46.39)		
Hemoglobin (g/L)	108.74±15.32	93.81±16.44	7.870	0.000
Blood uric acid (μmoL/L)	432.36±80.15	356.94±75.31	7.906	0.000
Albumin (g/L)	34.02±4.76	33.18±4.90	1.444	0.150
Protein breakdown metabolic rate (%)	0.71±0.28	0.67±0.16	1.318	0.188
KT/V	1.08±0.25	1.12±0.30	1.242	0.215

### *Analysis of factors influencing depression in the training set of peritoneal dialysis patients*

Gender (female = 1, male = 0), employment status (unemployed = 1, employed = 0), average monthly income (< 2000 Yuan = 1, ≥ 2000 Yuan = 0), cardiovascular complications (yes = 1, no = 0), cerebrovascular complications (yes = 1, no = 0), sleep disorders (yes = 1, no = 0), and continuous variables such as hemoglobin and blood uric acid were included in the logistic regression equation for analysis. The results showed that gender, employment status, average monthly income, cardiovascular complications, cerebrovascular complications, and sleep disorders are risk factors for depression in peritoneal dialysis patients (*p* < 0.05), while hemoglobin and blood uric acid are protective factors against depression in peritoneal dialysis patients (*p* < 0.05). See Table 3.

**Table 3:** Results of logistic regression analysis.

Factors	β	SE	Wald χ^2^	OR	95 % CI	p
Gender	0.937	0.390	5.753	2.551	1.187∼5.484	0.016
Working condition	1.329	0.401	10.997	3.777	1.722∼8.284	0.001
Monthly income	1.457	0.397	13.452	4.292	1.971∼9.350	0.000
Angiocardiopathy	1.641	0.452	13.214	5.162	2.131∼12.507	0.000
Cerebrovascular	2.085	0.578	13.023	8.048	2.593∼24.977	0.000
Sleep disorders	1.518	0.417	13.233	4.565	2.014∼10.346	0.000
Hemoglobin	−0.063	0.014	19.186	0.939	0.913∼0.966	0.000
Blood uric acid	−0.016	0.003	32.471	0.984	0.978∼0.989	0.000
Constant	9.355	1.771	27.891	7.971	‒	0.000

### *Development of the nomogram model*

**Fig. 2:**
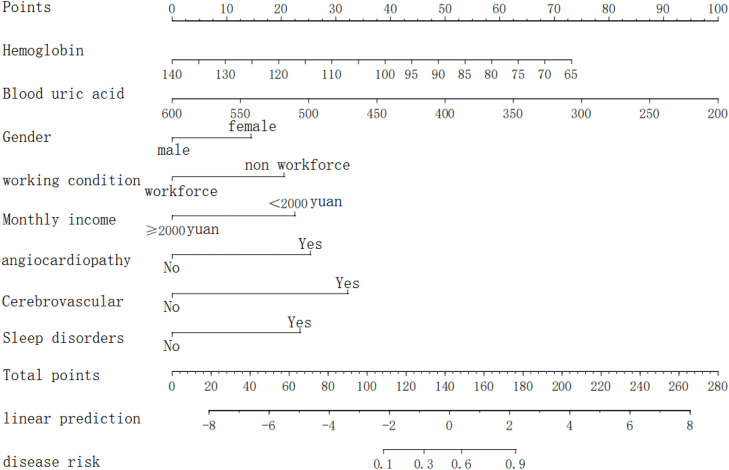
Nomogram for predicting the risk of depression in peritoneal dialysis patients.

Based on the results of logistic regression analysis, a nomogram model was constructed. The model for predicting the risk of depression in peritoneal dialysis patients included gender, employment status, average monthly income, cardiovascular complications, cerebrovascular complications, sleep disorders, hemoglobin, and blood uric acid. See Fig. 2.

### *Internal and external validation*

**Fig. 3:**
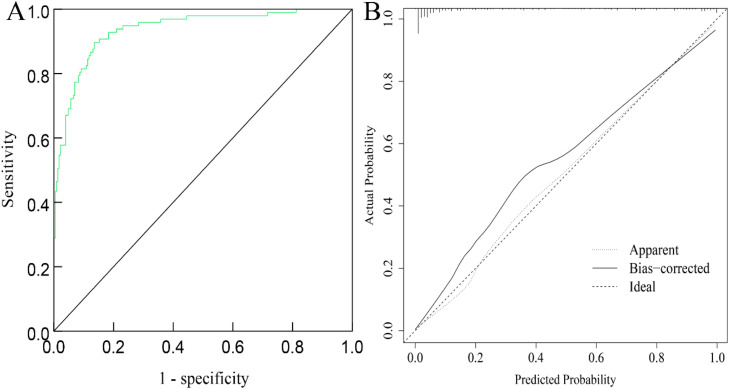
ROC curve (A) and calibration curve (B) of the nomogram for predicting depression in peritoneal dialysis patients on the training set.

**Fig. 4:**
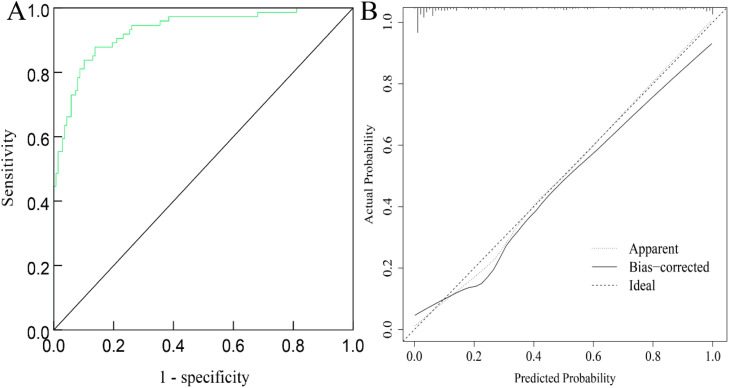
ROC curves (A) and calibration curves (B) of the nomogram for predicting depression in peritoneal dialysis patients on the validation set.

Regarding internal validation. the Hosmer-Lemeshow (H-L) goodness-of-fit test indicated a good fit of the nomogram model, χ^2^ = 3.570, *p* = 0.894. The ROC curve showed an AUC of 0.937 (95 % CI 0.907‒0.966), with a sensitivity of 89.78 % and a specificity of 86.52 %. Calibration curve analysis showed that the predicted probabilities closely matched the actual occurrence rates, as shown in Figs. 3A and 3B. In terms of external validation, the H-L goodness-of-fit test indicated a good fit of the nomogram model, χ^2^ = 5.140, *p* = 0.486. The ROC curve showed an AUC of 0.930 (95 % CI 0.894‒0.967), with a sensitivity of 87.86 % and a specificity of 86.24 %. Calibration curve analysis showed that the predicted probabilities closely matched the actual occurrence rates, as indicated in Figs. 4A and 4B.

### *Clinical utility assessment*

**Fig. 5:**
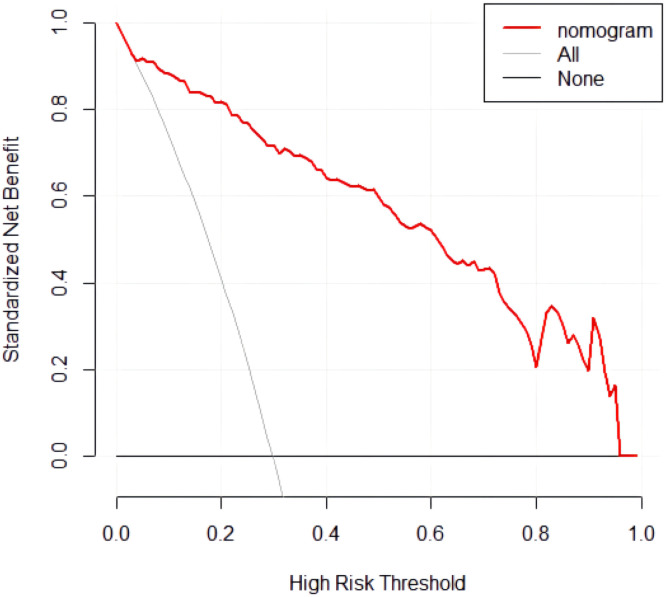
DCA curve of the nomogram.

The Decision Curve Analysis (DCA) was performed with the high-risk threshold on the x-axis and the net benefit on the y-axis. The high-risk threshold ranged from 0.05 to 0.97. The “None” line represents a scenario where none of the peritoneal dialysis patients develop depression, and the “All” line represents a scenario where all patients develop depression. The DCA curve results indicate that the nomogram model constructed in this study has good clinical utility, as shown in Fig. 5.

## Discussion

This study developed a novel predictive tool for estimating the risk of depression in peritoneal dialysis patients, incorporating eight baseline variables that include both traditional risk factors for depression and dialysis-specific factors. The final nomogram model demonstrated sufficient calibration, discrimination, and clinical utility.

The study revealed significant gender differences in the risk of depression among peritoneal dialysis patients, with female patients showing a significantly higher risk of depression than males. Previous research suggests that the gender difference in susceptibility to depression is caused by testosterone secretion, which has anti-anxiety and antidepressant effects.^10^^,^^11^ According to this study, the occurrence of depression in peritoneal dialysis patients is negatively correlated with average monthly income, and the proportion of depression among the employed population is relatively low. Many studies have demonstrated the impact of higher or lower income levels on depression, with low-income groups being more susceptible to depression.^12^^,^^13^ This may be due to the fact that low income affects psychosocial factors in these groups, with patients often experiencing long-term psychological stress due to concerns about affording high dialysis costs, thereby increasing their risk of depressive symptoms. In the study results, cardiovascular and cerebrovascular diseases are important influencing factors for the occurrence of depression in peritoneal dialysis patients. Previous studies believe that the expression of pro-inflammatory factors is related to the occurrence and development of cardiovascular diseases.^14^, ^15^, ^16^ However, it remains unclear whether the link between cardiovascular disease and depression is mediated by inflammation levels and immune mechanisms. Additionally, after cerebrovascular complications occur, ischemia and hypoxia in brain tissue and the release of a large amount of vasoactive substances.^17^^,^^18^ Patients with concurrent cardiovascular and cerebrovascular diseases often have limited mobility and may develop depression due to fear of disease recurrence or other complications. Dialysis patients, troubled by their illness and retaining a lot of uremic toxins in their body, often experience symptoms like itchy skin, leading to sleep disorders, daytime sleepiness, and inability to sleep at night, which can worsen their mood and trigger depression.^19^ Hemoglobin levels represent the state of anemia in the body, which is characterized by a decrease in red blood cells or hemoglobin in the bloodstream. The direct relationship between anemia and depression is not well understood.^20^ However, this study speculates that anemia can exacerbate various symptoms such as fatigue, irritability, and lack of concentration in dialysis patients. Given the significant physical pain these patients endure due to their condition, they are more likely to experience negative emotions, increasing their risk of depression. Uric acid, the end product of purine metabolism, is a strong antioxidant that scavenges free radicals in the body.^21^ Based on previous research on the biological function of uric acid, this study speculates that low levels of uric acid, which acts as a protective factor, may impair the body's ability to eliminate free radicals. The accumulation of large amounts of free radicals may cause oxidative damage to brain neurons, thereby increasing the risk of depression.

The identification of predictive factors and risk assessment are crucial for making effective medical decisions to prevent depression in peritoneal dialysis patients. For dialysis patients, multifactorial interventions have been proven to significantly reduce the risk of depression. Currently, there is a lack of individualized predictive tools for the risk of depression in peritoneal dialysis patients, both domestically and internationally. A nomogram, based on the total score of multiple predictive variables of an individual, allows clinicians to calculate the risk of an event occurring.^22^^,^^23^ In a nomogram, each variable is represented by a scale on a line, indicating its range of values, with the length of the line reflecting its contribution to the outcome event. Points in the nomogram correspond to individual scores for different values, and the total score is obtained by adding these individual scores. In developing a model for predicting depression in the peritoneal dialysis population, this study selected clinically simple and easily collectible variables, which enhances the clinical utility of the nomogram in this study. The nomogram developed in this study can estimate the probability of depression in peritoneal dialysis patients, helping clinicians make more favorable treatment decisions for patients. The nomogram calculates the probability of depression, allowing patients to be categorized into high-risk and low-risk groups. Policymakers should consider more comprehensive mental health screening for high-risk patients, particularly those who are women, unemployed, low-income, or have concurrent cardiovascular and cerebrovascular diseases, sleep disorders, low uric acid, and low hemoglobin levels. Efforts should be made to transition high-risk patients to low-risk to prevent the onset of depression.

However, it is important to acknowledge the limitations of this study. Firstly, apart from some variables measured during hospital examinations, all data in this study were derived from patient and family interviews, which may limit the accuracy of the data and somewhat impair the objectivity of the results. Secondly, due to the limited number of cases and sample sources, the inclusion of variables was subject to selection bias, and the study could not encompass all covariates related to peritoneal dialysis and depression, so some variables may have been overlooked. Additionally, the prevalence of depression might vary due to different assessment tools, and in the future, multiple scoring assessments for depression could be considered.

In summary, this study developed a new nomogram incorporating factors such as gender, employment status, average monthly income, cardiovascular complications, cerebrovascular complications, sleep disorders, hemoglobin, and blood uric acid. This tool can assess the risk of depression in peritoneal dialysis patients to a certain extent.

## Ethics approval and consent to participate

This study involving human participants was in accordance with the ethical standards of the Medical Ethics Committee of Zhuzhou Central Hospital (2023–02–013) and with the 1964 Helsinki Declaration. Obtaining the informed consent form of the patient or their guardian, and sign on the informed consent form. The study has been carried out in accordance with the STROBE Statement.

## Consent for publication

All authors give consent for publication.

## Authors’ contributions

Ming Yang: Project development; Data Collection; Data analysis; Manuscript writing, Xinhai Tang: Data collection; Data analysis, Yehua Fang: Project development; Data collection; Data analysis; Manuscript editing.

## Declaration of competing interest

The authors declare no conflicts of interest.
